# Cashew gum hydrogel as an alternative to minimize the effect of drought stress on soybean

**DOI:** 10.1038/s41598-024-52509-2

**Published:** 2024-01-25

**Authors:** Rafael Felippe Ratke, Alan de Sousa, Daniela Vieira Chaves, Fábio Luiz Zanatta, Ricardo Loiola Edvan, Heldeney Rodrigues Sousa, Edson Cavalcanti Silva-Filho, Josy Anteveli Osajima, Ariane Maria Silva Santos Nascimento, Jorge González Aguilera, Alan Mario Zuffo, Natielly Pereira da Silva, Paulo Eduardo Teodoro, Leilson Rocha Bezerra, Hebert Hernán Soto Gonzales, Luis Morales-Aranibar

**Affiliations:** 1 Agronomic Departament, Federal University of Mato Grosso do Sul (UFMS), Chapadão do Sul, Mato Grosso do Sul 79650-000 Brazil; 2 Agronomic Departament, Federal University of Piauí, Bom Jesus, Piauí 64900-000 Brazil; 3 Department of Animal Science, Agricultural Science Center, Federal University of Piauí, Teresina, Piauí 64049-550 Brazil; 4 Interdisciplinary Laboratory for Advanced Materials, LIMAV, Piauí, Federal University, Campus Universitário, MinistroPetrônio Portella, Teresina, Piauí 64049-550 Brazil; 5 Department of Crop Science, State University of Mato Grosso do Sul, Cassilândia, MS 79540-000 Brazil; 6 Agronomic Departament, State University of Maranhão, Campus de Balsas, Balsas, MA 65800-000 Brazil; 7 Veterinary Medicine Academic Unit, Campina Grande Federal University, Patos, Paraíba 58708-110 Brazil; 8 National Intercultural University of Quillabamba, Cusco, 08741 Peru; 9 National University of Moquegua (UNAM), Ilo, 18601 Peru

**Keywords:** Plant sciences, Environmental sciences

## Abstract

The use of hydrogels helpsthe production of plants in drought-stress environments. Thus, this work evaluated using different hydrogels to minimize drought stress in soybean cultivation. The treatments employed two different hydrogels, one already commercialized and the other produced with cashew gum (*Anacardium occidentale*), five levels (0, 30, 60, 120, and 240 mg pot^−1^) of the hydrogels, and two levels of drought stress in sandy soil. The growth and yield of soybeans and the levels of macro- and micronutrients in soybeans were evaluated.growth. The use of CG hydrogel promoted 12% increase in protein content in the seeds in the when soybean plants were subjected to drought stress. The levels of 30 mg pot^-1^, corresponding to 7.5 kg ha^−1^, improved the ’morphological and productive parametersof the soybeans. The increasing levels of hydrogel promoted the increase in P, K, Ca, Mg, and Fe and reduced S and Cu on an exponential scale. The use of cashew gum hydrogel increased the K and Ca contents in soybean seeds compared to commercial hydrogel.

## Introduction

Soybean (*Glycine max* L.) is the most widely cultivated oilseed in the world. In the 2021/2022 season, soybeans were grown on more than 40 million hectares in Brazil, producing115 million tons of grains and an average yield of 3539 kg ha^−1^^1^. The agricultural production potential of a region is related to climate and soil management^2^.

Most soils in Brazil are favorable for agricultural production^3^. However, some limitations may occur, including drought stress. In the Brazilian Cerrado region, rainfall irregularities occur during growth periods, causing a 15- to 30-day drought stress for soybean fields^4^. Sentelhas et al.^2^ reported that Bom Jesus, Piauí, Brazil, could produce 4734 kg ha^−1^of soybean per year; however, the actual yield is 2723 kg ha^−1^, with drought stress being responsible for 54% of this production loss. Drought stress can be corrected with irrigation. However, sprinkler irrigation systems are expensive and consume a great amount of water, making the technique unfeasible for large cultivation areas^5^.

Technologies are neededto reduce the effects of drought stress^6^. One alternative is using hydro-absorbent polymers known as hydrogels^7^. The hydrogels absorb water and make it available when the environment has higher osmotic pressure^8^. Azevedo et al.^9^ reported that hydrogels are based on polyacrylamide, but several hydro-retentive products, such as cashew gum, can also be used^10^. Additionally, mixed or pure with polyacrylamide, chitosan, and alginate are used to make hydrogels^11^. Fertilizer-associated hydrogels can slowly release of nutrients to the plant, which can reduce fertilizer application operations during plant cultivation^12^.

Natural polymers, such as gums and cellulose, are increasingly studied for their potential use in agriculture^13^. These polysaccharides can be modified by crosslinking chemical and physical changes to improve their processability and reactivity^14^. Copolymerization has been identified as a useful technique for producing advanced materials with applications in agriculture^15^. Hydrogels produced using this method have shown potential in combating crop pathogens, minimizing nutrient loss in fertilization, improving productivity, and increasing the efficiency of agrochemicals^16^. Several protected technological inventions have been developed to manufacture superabsorbent hydrogels using natural polymers, highlighting their importance in developing new materials with potential applications in agriculture^17–19^.

The cashew tree (*Anacardium occidentale* L.) is a native fruit tree widely cultivated in northeastern Brazil, and its nut and pseudo fruit are economically exploited^20^. Cashew gum (CG) can also be extracted from the tree through periodic incisions in the trunk and branches of the tree^20^. CG is used in the chemical and pharmaceutical industry for its flexibility in chemical modification, hydrophilic character, low cost, biocompatibility, biodegradability, and applicability^20^. The abundance and ease of obtaining CG represent an opportunity to produce biodegradable hydrogels of renewable natural material^10^.

Hydrogels are used on a large scale in eucalyptus and pine forest plantations to ensure the survival of seedlings in the field^21^. The efficiency of using hydrogels has been proven in several scientific trials^22,23^. However, there are no reports of its efficiency in developing and producing annual crops such as soybeans.Hydrogels could eliminate the need to exclusively grow transgenic drought-resistant soybeans, making non-transgenic soybean production possible in drought susceptible regions.

Thus, we hypothesize that the cashew-based hydrogel will promote soybean growthand yield similar toa polyacrylamide-based hydrogel in 15-day drought stress. This research aimed to evaluate the yield parameters and nutrient contents in soybean seeds under simulated drought stress using different sources and levels of hydrogels.

## Materials and methods

### Trial conditions

**Figure 1:**
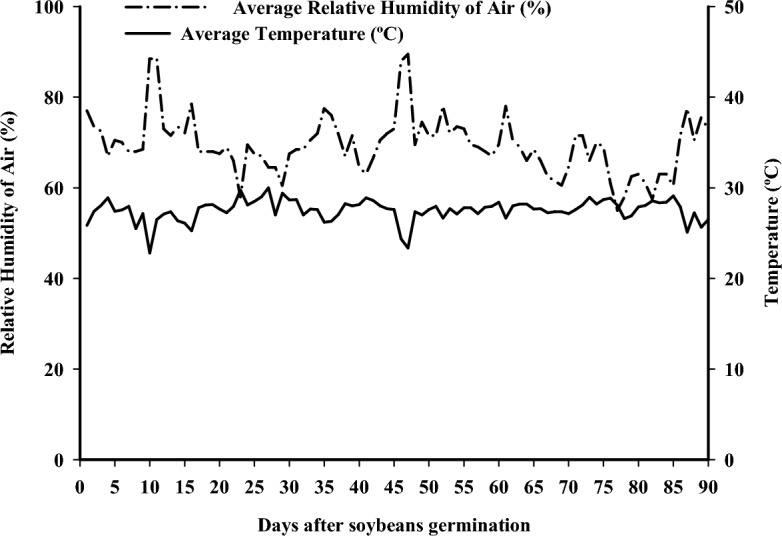
Average humidity data, and temperature during the soybean cultivation period, soybean planting on 02/18/2019. Source: Weather station installed at UFPI. Bom Jesus, Piauí, Brazil.

The trial was conducted in a vegetation house located at the Federal University of Piauí, Campus Professora Cinobelina Elvas (CPCE), in Bom Jesus, Piauí, Brazil (09° 04’S and 44° 21’W, and 277 m of the ASL) in 2019. According to the Köppen classification, the regional climate is Bsh^24^. The humidity data and temperature in the environment during soybean cultivation are described in Fig. 1. All methods were carried out following relevant institutional, national, and international guidelines and legislation.

### Experimental design and hydrogel materials

The experimental design was entirely randomized in a 2 × 2 × 5 factorial scheme with four repetitions. The factors were two levels of drought stress (with and without), two types of hydrogels [Hydroplan– E.B. commercial polyacrylamide-based hydrogel (H1) and cashew gum-based hydrogel with 5% K_2_HPO_4_ (H2)] and five levels of hydrogel (0, 30, 60, 120, and 240 mg pot^-1^), corresponding to 7.5, 15, 30 and 60 kg ha^−1^, respectively.

H2 was developed in the Material Science Laboratory of the Federal University of Piauí (LIMAV) from cashew gum isolate ina polymerization reaction with K_2_HPO_4_ and acrylamide,as described by Souza^10^. H2 has a swelling capacity of 1100 g H_2_O g^−1^ hydrogel. H1 fertilized with P and K (Table 1) has a swelling capacity of 6000 g H_2_O g^−1^ of hydrogel.

**Table 1:** Supplemental P and K fertilization in the commercial hydrogel trials.

Hydrogel		MAP (52% P_2_O_5_)		KCl^2^ (60% K_2_O)	
mg pot^−1^	kg ha^−1^	kg ha^−1^	kg pot^−1^	kg ha^−1^	mg plot^−1^
30	7.50	0.00	0.00	0.00	0.00
60	15	0.25	0.98	1.83	7.33
120	60	0.98	3.92	5.50	22.00
240	120	1.96	7.85	7.33	29.33

### Experimental conditions

The soil used was collected in a native area of the Cerrado biome at a depth of 0–0.20 m, classified as an atypical Oxisol with a loamy-sandy texture after collection. Chemical and physical attributes were determined according to Teixeira et al.^25^. In the soil analysis, the following values were found: pH in H_2_O = 4.6; H + Al = 4.62 cmol_c_ dm^−3^; Al^3+^  = 0.35 cmol_c_dm^−3^; Ca^2+^  = 0.08 cmol_c_dm^−3^; Mg^2+^  = 0.02 cmol_c_ dm^−3^; K = 9.2 mg dm^−3^; P = 5.73 mg dm^−3^; sum of bases = 0.12 cmol_c_ dm^−3^; cation exchange capacity in pH 7.0 = 4.74 cmolc dm^−3^; Ca + Mg + Ksaturation (%) = 2.61; aluminum saturation (%) = 74.46; Cu = 0.86 mg dm^−3^; Fe = 0.55 mg dm^−3^; Mn = 4.37 mg dm^−3^; Zn = 48.92 mg dm^−3^; Organic Matter = 6.1 g kg^−1^; Clay = 197 g kg^−1^; Silt = 4 g kg^−1^; and Sand = 799 g kg^−1^.

The soil requires acidity correction for good soybean cultivation. The ’2.42 t ha^−1^ or 1.21 g kg^−1^ soil (35% CaO and 6% MgO) of limestone was used to increase the soil base saturation by 50%^15^. The soil was fertilized with 1.76 g ha^−1^ of simple superphosphate (18% P_2_O_5_) and 0.33 g ha^-1^ of KCl (60% K_2_O), corresponding to 444 kg ha^−1^ and 83 kg ha^−1^, respectively, for soybean planting following the recommendation for soybean cultivation^26^. After liming and fertilization, the soil was homogenized and placed in each pot. The pots had a capacity of 10 L and were filled with 8 kg of soil.

Soil moisture was determined before irrigation to implement drought stress. Thus, 52.21 g of soil was weighed on an analytical balance (0.001 g) and then put into the circulating oven for 24 h at a temperature of 105 °C. The sample was reweighed to 52.09 g, and 0.22% moisture was obtained. After determining the soil moisture, we calculated the soil water capacity (SWC) in four pots, adding 2000 ml of distilled water to the pot for a period of 48 h and retaining 1654 ml of water, considering this value to be 100% of the SWC The potting soils were irrigated to maintain 80% of the SWC.

At the R1 stage, the bloom of soybeansand soybeans in the drought-stress trials were not irrigated for 15 days. The drought stress during the harvest period in productive regions was simulated, and soybeans were irrigated again after this period. In the no-drought-stress trials, soybeans continued to receive normal irrigation during this 15-day period.

Because the cashew-based hydrogel (H2) has P and K in its formulation, it was necessary to supplement P and K in the commercial hydrogel (H1). We applied supplementary fertilization with phosphorus (P) and potassium (K), as shown in Table 1, at soybean planting.

Sowing was performed with soybean variety BRS 7780 iPRO with a cycle of 102 daysfrom the affiliated institute on 02/18/2019. The soybean seeds were inoculated with *Bradyrhizobium japonicum* bacteria at 100 ml for every 50 kg of soybean seed. At planting, the respective hydrogels and their specific levels were introduced at a depth of 0.05 m and then covered with a layer of soil, thus depositing the seed to a depth of 0.02 m. Twenty days after the emergence (DAE) of the seedlings in vegetative stage V3, the plants were thinned, leaving only one plant per pot, and more vigorous plants were selected.Micronutrient fertilization was performed by applying 8 ml pot^−1^ of a micronutrient solution containing B = 0.3, Cu = 0.02, Fe = 2.0, Mn = 0.4, Mo = 0.06, and Zn = 0.06 mg L^−1^ at 15, 30, and 45 DAE.

### Plant growth and yield

At harvest time, 102 days after soybean planting, the root volume (RV), root dry mass (RDM), first pod height (FPH), number of pods (NP), number of seeds (NS), number of seeds per pod (NSP), and seed mass (SM) were measured.

RV and RDM were performed by washing the roots. The roots were separated from the aboveground part of the plant and washed to remove soil. Later, the roots were placed in a 1000 ml cylinder with 500 ml of water, and the displacement of the water column in the cylinder considered the volume of the roots, transforming the value from ml to cm^3^ per pot (1 cm^3^ = 1 ml). The roots measured in the measuring cylinder were dried in a forced ventilation oven at 65 °C for 72 h and later weighed on an analytical scale (0.001 g) to obtain the MDR. The first pod was inserted in the soybean stem with the help of a ruler graduated in cm. The pod and seed were measured by removing them from the soybean plants, counting the pods and seeds, and weighing the seeds on an analytical balance (0.001 g), and the seed weight was standardized to a humidity of 13%.

The soybeans were dried in an oven at 65 °C for 72 h and milled in a Willey-type mill. After milling, the concentrations of N, P, K, Ca, Mg, and S in the seeds were determined, as proposed by Silva^16^. The Kjeldahl method analyzed the ’protein contentof soybeans, as described by Detmann et al.^17^.

### Statistics analysis

The analysis of the variance of the results was performed using Rbio software^18^. For the qualitative treatments (drought stress and sources) that had a significant effect, the Scott‒Knott test of means was done (p < 0.05). Regression analysis was used for the levels of hydrogels, and significance equations (F test, p < 0.05) were adjusted according to the highest coefficients of determination.Pearson’s correlation coefficients were evaluated between the productive parameters of soybean and nutrients in soybean seeds.

## Results and discussion

The parameters root volume, height of the first pod, number of pods, number of seeds, number of seeds per pod, and seed mass of soybeans presented isolated effects for hydrogel levels (Table 2). A significant interaction was observed between drought stress and hydrogel source on the root volume, root mass, number of seeds per pod, and soybean protein content. Soybean yield parameters showed no effects of the interaction between drought stress, hydrogel sources, and levels.

**Table 2:** Analysis of variance and significance by F test for yield and protein parameters in soybean as a function of water deficit and different sources and levels of hydrogel.

Trials	RV	RDM	FPH	NP	NS	NSP	MS	PR
	Calculated F values^+^							
Drought stress (D)	1.58	9.59*	1.39	0.28	0.02	1.35	1.69	1.06
Hydrogels (H)	0.61	0.87	0.1	1.35	0.76	0.34	0.11	1.69
Levels (L)	2.59*	0.78	6.18*	21.93*	29.34*	5.29*	16.98*	0.79
D*H	4.56*	10.81*	1.39	0.15	0.02	4.23*	0.39	7.81*
D*L	0.49	0.45	0.84	0.44	0.47	0.98	1.29	1.61
H*L	1.89	0.69	0.7	0.82	0.41	1.65	0.47	0.36
D*H*L	0.06	1.73	0.68	0.33	0.61	1.41	0.44	1.36
C.V. (%)	57.06	49.84	10.25	26.37	24.05	26.92	27.14	13.36

*RV* Root volume, *RDM* root dry mass, *FPH* first pod height, *NP* number of pods, *NS* number of seeds, *NSP* number of seeds per pod, *SM* seed mass, *PR* Protein.

^+^F test values in analysis of variance.

*Significant (p < 0.05).

Drought stress reduced the root mass of soybean by 30% compared to the treatment with normal irrigation (Table 3). Root mass was 44% higher using commercial hydrogel than CG hydrogel when soybean plants were subjected to drought stress. The average NSP did not show significant differences for the integrated factors of drought stress and hydrogel sources. The use of CG hydrogel promoted 12% increase in protein content in the seeds in the when soybean plants were subjected to drought stress.

**Table 3:** Mean values for yield and protein parameters in soybean as a function of water deficit and different sources and doses of hydrogel.

Trials	RV	RDM	NSP	PR
	cm^3^ pot^−1^	g pot^−1^	Seeds Pods^−1^	%
Without drought stress	5.30*	3.21^A^	2.42	40.36
Commercial hydrogel (H1)	2.87^aA^	2.84^aA^	2.42^a^	41.25^aA^
Cashew hydrogel (H2)	5.72^aA^	3.56^aB^	2.33^a^	39.47^aA^
With drought stress	4.51	2.26^B^	2.37	39.13
Commercial hydrogel (H1)	5.42^aA^	2.90^aA^	2.42^a^	36.71^bB^
Cashew hydrogel (H2)	3.60^bB^	1.61^bB^	2.33^a^	41.57^aA^

*Means followed by the same lowercase letter for hydrogel sources and uppercase for dose stress in the column do not differ by the Scott‒Knott test (p < 0.05).

Hydrogels alleviated drought stress and thus promoted conditions for the growth and production of soybean plants. When comparing the water retention of the cashew gum-based hydrogel with that of other hydrogels, the same behavior was found for polyvinylpyrrolidone (PVP)/carboxymethyl cellulose (CMC)-based hydrogels. Chitosan,-g-poly(acrylate),and guar gum-g-poly(acrylate) were efficient in retaining water in soil^12,27^. Thus, using hydrogels based on CG under drought-stress conditions favors the growth and production of soybeans. The supply of water and nutrients to the plant must be adequate to meet needs, and climatic events such as drought can affect the physiology of the soybean and decrease its productive capacity^28^.

The ’chemical composition of the hydrogelsinterfered with the plants’ absorption and desorption of water and nutrients^29^. However, the presence of ions in the soil does not reduce the ability of the chitosan-based hydrogel to absorb water, but it decreases the release of K^12^. Thus, water adsorption on the cashew gum-based hydrogel was lower due to the addition of potassium and phosphorus ions^10^.

The water adsorption of PVP and CMC-based hydrogels is lower in solutions containing fertilizers than in deionized water^27^. Narjaryand Aggarwal^30^ reported that the available water content increased and root penetration resistance decreased when using a 5 kg ha^−1^ level of hydrogel, which favors root growth.

**Figure 2:**
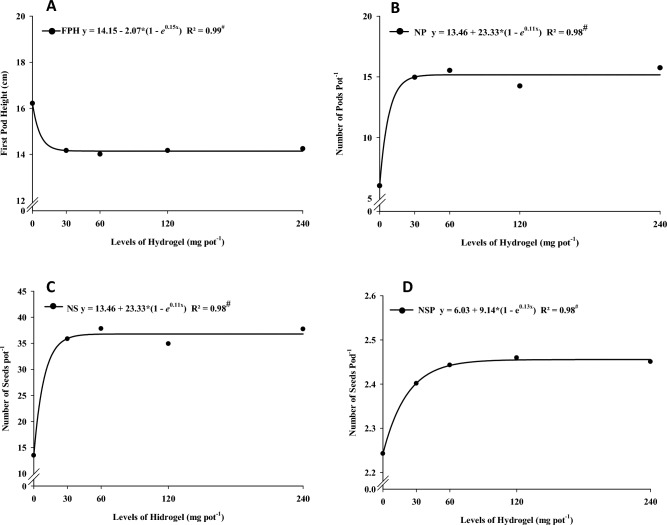
Exponential regression for first pod height (**A**), number of pods (**B**), number of seeds (**C**), and number of seeds per pod (D) as a function of hydrogel dose. ^#^Significant regression equationby the F test (p < 0.05).

**Figure 3:**
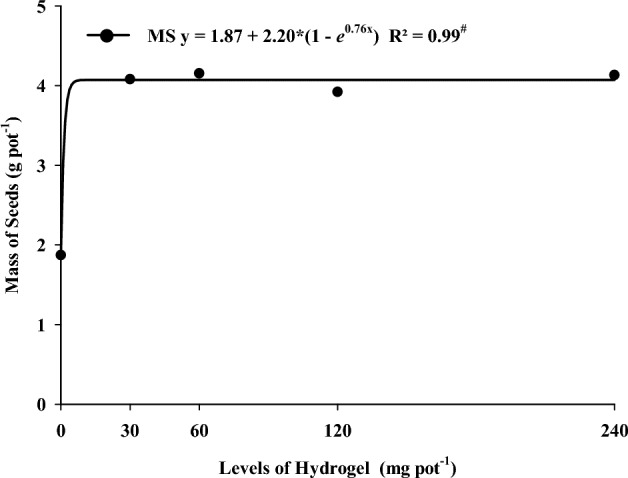
Exponential regression for seed mass as a function of hydrogel dose. ^#^Significant regression equation by the F test (p < 0.05).

The first pod height of soybean decreased with increasing hydrogel levels (Fig. 2A). The number of pods, the number of seeds, and the number of seeds per pod increased with higher levels of hydrogel (Fig. 2B,C,D). Using levels above 30 mg pot^−1^ of hydrogel promoted the highest soybean seed mass regardless of drought stress (Fig. 3).

The level of 30 mg pot^−1^, which corresponds to 7.5 kg ha^−1^, was sufficient to meet the ’water requirements of the plant and favor the soybean production parameters. ElbarbaryandGhobashy^31^ described that using PVP-based and CMC-based hydrogels prepared by gamma radiation on soil promoted the growth of corn plants when compared to the control treatment. The use of hydrogels in agriculture favors the growth and production of several crops in water-restricted environments^8^.

The recommendation of levels of hydrogels in agriculture depends on the soil type, sandy, clay, silty, or organic soil. Narjary and Aggarwal^30^ described that using hydrogels based on acrylamide and cellulose derivatives at 0.7% is sustainable in sandy soils, promoting a water regime interval of up to 22 days. In clayey and organic soil, the critical water levels for crops were reached in a shorter time. However, in our study, using hydrogels at levels of 30 mg pot^−1^, corresponding to 0.0018%, was sufficient to favor the productive parameters of soybean under 15-day drought stress.

The concentrations of S and Mn in soybeans were influenced by the interaction of drought stress, different sources, and levels of hydrogel (Table 4). The K, Ca, Cu, Fe, Mn, and Zn levels in soybeans showed an isolated effect for the hydrogel source. The hydrogel levels significantly influenced the contents of P, K, Ca, Mg, S, Cu, Fe, and Mn in soybeans. The effects of the interaction between drought stress and hydrogel sources on the N contents in soybean seeds were observed.

**Table 4:** Analysis of variance for macro- and micronutrients in soybean seeds as a function of drought stress and different sources and levels of hydrogel.

Trials	N	P	K	Ca	Mg	S	Cu	Fe	Mn	Zn
	Calculated F values^+^									
Drought stress (D)	1.06	0.34	1.33	3.33	0.89	6.14*	0.1	0.45	5.87*	0.37
Hydrogels (H)	1.69	0.21	6.89*	5.99**	3.48	3.34	7.97*	23.13*	53.99*	7.03*
Levels (L)	0.79	9.18*	2.99**	7.35*	7.96*	23.49*	8.46*	2.62*	4.29*	0.89
D*H	7.81*	1.08	0.01	0.01	2.2	0.09	0.05	1.57	0.46	2.11
D*L	1.62	0.21	1.19	1.27	0.55	1.29	1.44	1.66	2.82	0.29
H*L	0.35	0.26	1.56	0.11	0.86	0.75	0.7	2.42	1.46	0.08
D*H*L	1.36	1.05	2.11	1.7	0.16	3.23*	0.4	1.24	6.28*	0.95
C.V. (%)	13.36	24.40	14.61	18.54	23.23	16.35	51.88	59.88	28.50	20.6

^+^F test values in analysis of variance.

**Significant (p < 0.05).

The lowest N contents in soybean seeds were observed using commercial hydrogel and drought stress (Table 5). Using cashew hydrogel without drought stress decreased the P content in soybeans. The K and Ca contents in soybean seeds were higher using cashew hydrogel. The use of commercial hydrogels proportionally increases the Zn content in soybeans.

**Table 5:** Average levels of macro- and micronutrients in soybean seeds as a function of drought stress and different sources and levels of hydrogel.

Trials	N	P	K	Ca	Zn
	g kg^−1^				mg kg^−1^
Without drought stress	64.58	5.11	21.62^a^	4.38^a^	31.60
Commercial hydrogel (H1)	66.00^aA^	5.33^aA^	20.72	4.59	34.64
Cashew hydrogel (H2)	63.16^aA^	4.90^aB^	22.52	5.07	28.56
With drought stress	62.62	5.28	20.82^a^	4.48^a^	32.5
Commercial hydrogel (H1)	58.73^bB^	5.21^aA^	19.9	4.255	33.39
Cashew hydrogel (H2)	66.51^aA^	5.36^bB^	21.74	4.718	31.60
Average H1	62.36	5.27	20.31^b^	4.42^b^	34.01^a^
Average H2	64.83	5.13	22.13^a^	4.89^a^	30.08^b^

*Means followed by the same lowercase letter for hydrogel sources and uppercase for dose stress in the column do not differ by the Scott‒Knott test (p < 0.05).

The types of hydrogels influence the nutrient contents in soybeans. The release of ions by polymer hydrogels depends on diffusion mechanisms and is generally related to adsorption capacity^10^. The phosphorus in H2 has a slower release than the potassium ion, which may have decreased the uptake and concentration of P in the soybean seed.

**Figure 4:**
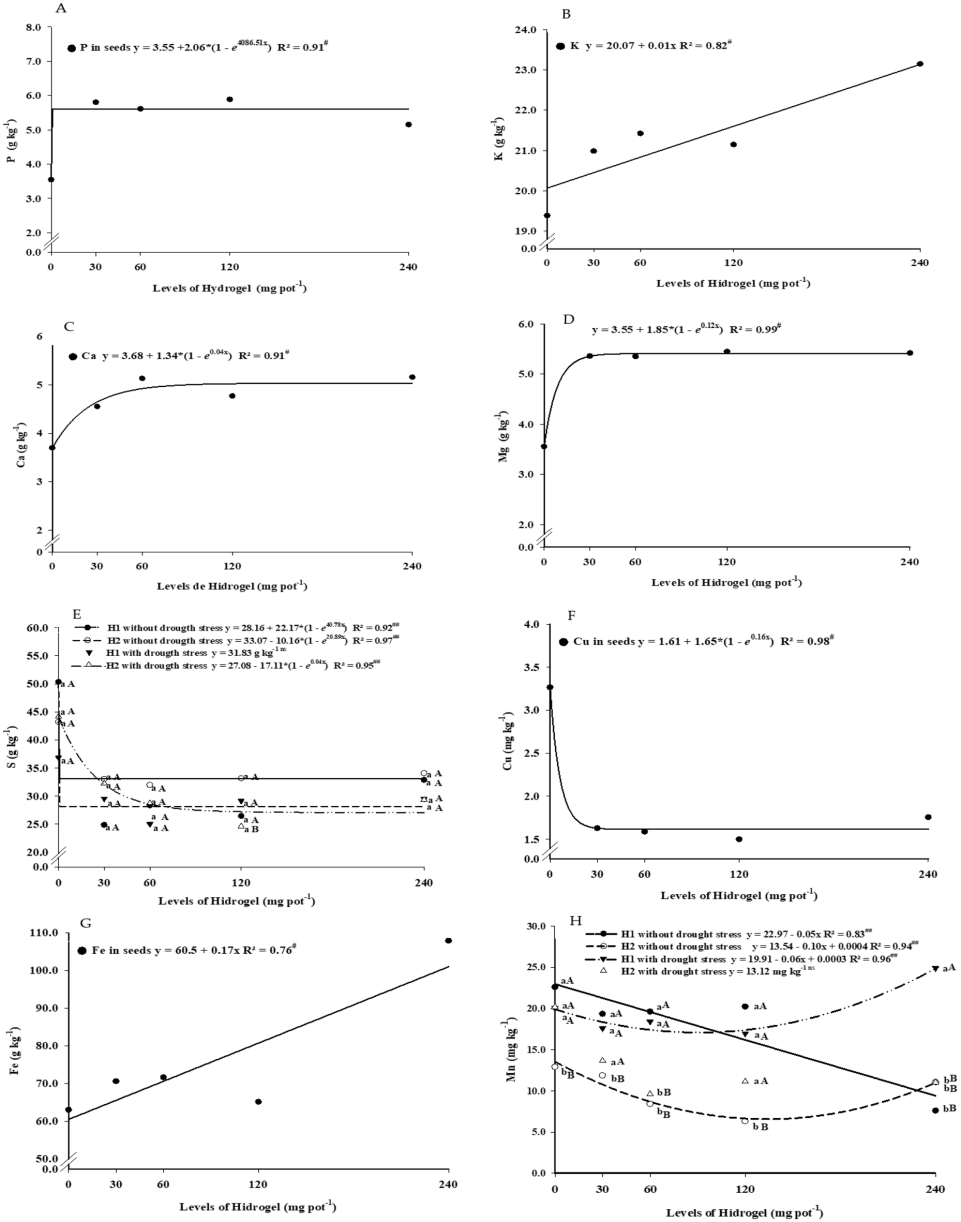
Averages of P (**A**), K (**B**), Ca (**C**), Mg (**D**), S (**E**), Cu (**F**), Fe (**G**), and Mn (**H**) contents as a function of hydrogel doses. ^#^Significant regression equation by the F test (p < 0.01); and ^##^Significant regression equation, by the F test (p < 0.05). Symbols followed by the same lowercase letter do not differ for hydrogel sources by Scott‒Knott test of means (p < 0.05). Symbols followed by the same uppercase letter do not differ for water deficit by the Scott‒Knott test of means (p < 0.05).

The hydrogel levels provided different responses for nutrients in soybeans (Fig. 4). The P, Ca, and Mg contents in soybeans showed significantly increasing exponential regression for increasing levels of hydrogel (Fig. 4A,C,D). The higherlevels of hydrogel increased the K and Fe contents in soybeans, providing significant linear regression (Fig. 4B and G). The S and Cu contents were reduced in soybeans as a function of hydrogel levels (Fig. 4E and F). Only the trial with H1 with drought stress did not proportion exponential to least significant regression for S contents in soybeans as a function of hydrogel levels. The lowest S content in soybeans was 24.63 mg kg^−1^, observed in the H2 trial with drought stress.

The Mn contents in soybeans showed linear regression for levels of H1 in the trial without drought stress, so when the levels of hydrogel increased, the Mn contents decreased (Fig. 4H). However, the trials with H2 without drought stress and H1 with drought stress showed significant polynomial regression for Mn contents in soybeans as a function of hydrogel levels. The lowest Mn contents in soybeans were observed in H2 treatments without drought stress in all levels of hydrogel, and the same result was found for H1 at 240 mg pot^-1^ in the trial without drought stress.

At the level of 120 mg pot^−1^ of H1, the highest value of Mn in soybeans was observed (16.96 mg kg^−1^) and was 62% higher than the lowest value of Mn at this level observed in the trial of H2 in the trial without drought stress. Thus, the trial with the 240 mg pot^−1^ level of H1 with drought stress had the highest value of Mn in soybeans (24.91 mg kg^−1^), which was 70.5% higher than the trial with H1 without drought stress.

Hydrogels can retain and slowly release nutritional elements. Chandrika^13^ found that poly(vinyl alcohol), poly(vinyl alcohol)/chitosan, and chitosan-based hydrogels coated with NPK. Fertilizers when immersed in water for 30 days released 84, 12, and 36 mg L^−1^ of the nutrients N, P, and K, respectively. Elbarbary and Ghobashy^32^ described that PVP- and CMC-based superabsorbent hydrogels prepared by gamma radiation promoted N and P desorption in soil, with N desorption being ten times greater than P release. Elbarbary and Ghobashy^31^ reported that hydrogels made from chitosan (C.S.) and 2-hydroxyethyl methacrylate (HEMA) modified by phosphorylation are efficient in adsorbing Ca(II), Cu(II), and Zn(II) in aqueous solutions.

The hydrogel based on cashew gum and polyacrylamide affectedtheadsorption and desorption of ions in the soil, which influenced the availability of nutrients for soybean and changed the chemical composition and the protein content in the seed. Hydrogels can be degraded by microorganisms and ions existing in the soil, which influences the release of nutrients in the soil^33^. However, the slowrelease of water and nutrients depend on the type of material used to manufacture the hydrogel because there are made of easy soil degradation materials, such as chitosan^12^. Thus, the release of chemical elements in the soil and their absorption and accumulation in soybeans depends on the type of hydrogel used^10,14,16,17,19,33^.

**Figure 5:**
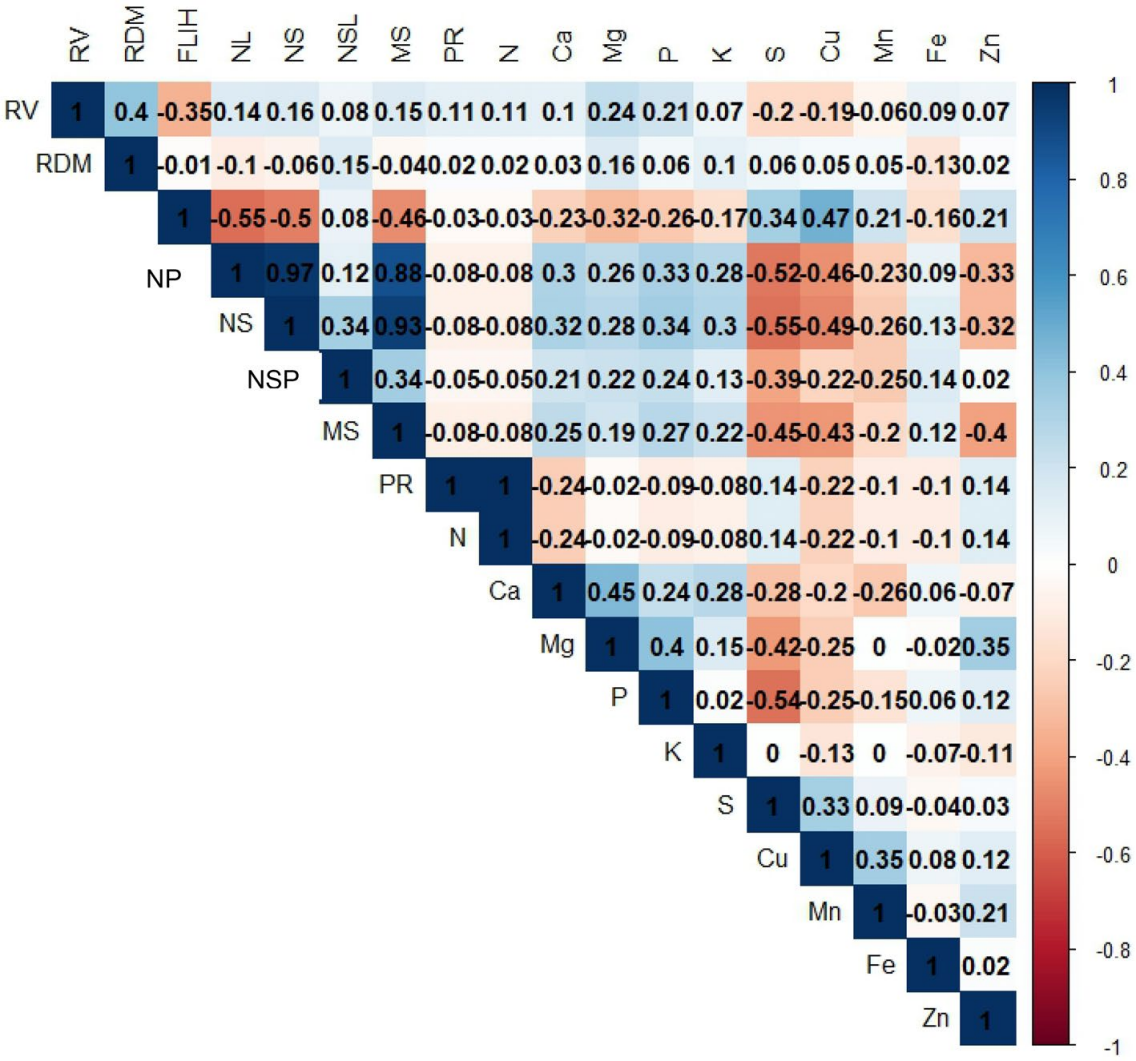
Pearson correlations averages of the *RV* Root volume, *RDM* root dry mass, *FPIH* first pod height, *PL* number of pods, *NS* number of seeds, *NSP* number of seeds per pod, *SM* a seed mass, *PR* Protein, and nutrients of seeds: N, Ca. Mg, P, K, S, Cu, Mn, Fe, and Zn.

The NS, NSP, and MS parametersas well as N and PRshow a strong correlation (Fig. 5). However, the correlations between soybean production parameters and nutrients in soybean seeds were low when using different sources and doses of hydrogels in the dry season and without the dry season. The NPK levels in soybean seeds are correlated with the level of weeds in competition with soybean^34^. In this sense, the management of soybean cultivation influences the levels of nutrients in soybean seeds. Li et al.^35^ found that the levels of protein and N in soybean grains are more correlated, especially with N in the form of NH_4_.

## Conclusions

The hydrogels, regardless of their composition, reduced the effect of drought stress on soybean growth and yield.The slow release nutrients to seeds of soybeans is derived from the structure of the hydrogels and materials used. The tested hydrogels can be used to minimize the effect of drought in arid regions to cultivate soybean, with the cashew gum hydrogel being a sustainable material.

## Data Availability

The datasets used and/or analyzed during the current study are available from the corresponding author upon reasonable request.
